# Effects and neuroprotective mechanisms of vagus nerve stimulation on cognitive impairment with traumatic brain injury in animal studies: A systematic review and meta-analysis

**DOI:** 10.3389/fneur.2022.963334

**Published:** 2022-09-27

**Authors:** Han Zhang, Chun-liu Li, Yun Qu, Yu-xuan Yang, Juan Du, Yu Zhao

**Affiliations:** ^1^Department of Rehabilitation Medicine, West China Hospital of Sichuan University, Chengdu, China; ^2^Department of Rehabilitation Medicine, Second Clinical Medical College of North Sichuan Medical College, Nanchong Central Hospital, Nanchong, China; ^3^College of Rehabilitation Medicine, West China Hospital of Sichuan University, Chengdu, China; ^4^Sichuan Provincial Key Laboratory of Rehabilitation Medicine, Sichuan University, Chengdu, China

**Keywords:** vagus nerve stimulation, cognition, traumatic brain injury, systematic review, meta-analysis

## Abstract

**Introduction:**

Cognitive impairment is the main clinical feature after traumatic brain injury (TBI) and is usually characterized by attention deficits, memory loss, and decreased executive function. Vagus nerve stimulation (VNS) has been reported to show potential improvement in the cognition level after traumatic brain injury in clinical and preclinical studies. However, this topic has not yet been systematically reviewed in published literature. In this study, we present a systematic review and meta-analysis of the effects of VNS on cognitive function in animal models of TBI and their underlying mechanisms.

**Methods:**

We performed a literature search on PubMed, PsycINFO, Web of Science, Embase, Scopus, and Cochrane Library from inception to December 2021 to identify studies describing the effects of VNS on animal models of TBI.

**Results:**

Overall, nine studies were identified in animal models (36 mice, 268 rats, and 27 rabbits). An analysis of these studies showed that VNS can improve the performance of TBI animals in behavioral tests (beam walk test: SMD: 4.95; 95% confidence interval [CI]: 3.66, 6.23; *p* < 0.00001) and locomotor placing tests (SMD: –2.39; 95% CI: –4.07, –0.71; *p* = 0.005), whereas it reduced brain edema (SMD: –1.58; 95% CI: –2.85, –0.31; *p* = 0. 01) and decrease TNF-α (SMD: –3.49; 95% CI: –5.78, –1.2; *p* = 0.003) and IL-1β (SMD: –2.84; 95% CI: –3.96, –1.71; *p* < 0.00001) expression level in the brain tissue. However, the checklist for SYRCLE showed a moderate risk of bias (quality score between 30% and 60%), mainly because of the lack of sample size calculation, random assignment, and blinded assessment.

**Conclusion:**

The present review showed that VNS can effectively promote cognitive impairment and neuropathology in animal models of TBI. We hope that the results of this systematic review can be applied to improve the methodological quality of animal experiments on TBI, which will provide more important and conclusive evidence on the clinical value of VNS. To further confirm these results, there is a need for high-quality TBI animal studies with sufficient sample size and a more comprehensive outcome evaluation.

**Systematic review registration:**

https://www.crd.york.ac.uk/prospero/display_record.php?ID=CRD42021290797, identifier: CRD42021290797.

## Introduction

Traumatic brain injury (TBI) is one of the most common causes of disability and social withdrawal in young adults (1). TBI-induced cognitive impairment is greatly associated with reduced quality of life (2). People with TBI have cognitive impairments, including characteristics such as poor planning, distractions, memory deficits, and difficulty in decision-making and verbal expression (3). In addition, brain damage may affect the individual personality and social behavior of patients, which are closely related to cognitive function (4). Current therapeutic strategies for cognitive dysfunction in traumatic brain injury consist of cognitive rehabilitation, pharmacotherapy, and noninvasive brain stimulation (NBS) (5). Cognitive rehabilitation involves repeated standardized cognitive tests of increasing difficulty targeting specific cognitive domains (e.g., selective attention and memory for new information) and the use of assistive technology (AT), calendars, electronic memory devices, alarms, or reminders as compensatory techniques. However, cognitive rehabilitation is best suited for well-motivated, functionally independent individuals with mild to moderate cognitive impairment (6). In addition, amantadine may improve attention, visuospatial function, and executive function in patients with TBI (7), and a few studies have reported the potential use of methylphenidate in patients with TBI. But there is insufficient evidence to support its use in patients with moderate to severe brain injury (8, 9). NBS includes transcranial magnetic stimulation (TMS) and transcranial direct current stimulation (tDCS), and studies have suggested that NBS may have positive changes in mood, visuospatial function, language, working memory, and/or executive function. However, this evidence is largely theoretical and further research is needed to establish a clear role for NBS in TBI. Therefore, there is an urgent need to investigate an effective approach for correcting cognitive impairment to minimize the adverse effects on patients with TBI.

Recently, vagus nerve stimulation (VNS) has been suggested as a promising tool for improving cognitive impairment (10, 11). Over the past few decades, the modulation of vagal nerve function has shown clinical efficacy for the treatment of conditions, such as epilepsy, depression, and migraine (12). Studies have shown that VNS can strengthen cognitive function, particularly executive function, in healthy individuals (13–15). Consequently, changes in executive function are associated with VNS increasing the activity of brainstem nuclei that may be involved in the pathophysiology of Alzheimer's disease (AD) (16); this results in increased circulation of monoamine neurotransmitters (norepinephrine [NE], 5-hydroxytryptamine, and dopamine). These transmitters affect the performance of executive functions. Gains in NE concentration may also increase functional connectivity in the hippocampus, amygdala, and prefrontal cortex, consequently contributing to memory strength (17–19). Animal studies have shown that VNS enhances learning and memory formation by increasing long-term potentiation, synaptic plasticity, and upregulation of endogenous neural stem cells (20, 21). On the one hand, under acute/hyperacute conditions (primary injury sustained from the initial traumatic force), VNS may improve brain metabolism and intracranial pressure by alleviating spreading depolarization phenomena (22), excitotoxicity, and inflammation (23–25). On the other hand, under chronic situations, VNS may upregulate plasticity and adrenergic and excitatory neurotransmitters to promote cognitive and motor recovery (26–28). Thus, all of these mechanisms appear to underlie the potential VNS-mediated cognitive recovery in TBI.

Despite several reports suggesting the positive effects of VNS on different cognitive impairments, the specific mechanisms of action have rarely been explored in TBI studies. As behavioral studies and morphological changes in the brain are indispensable when studying the effects of VNS on cognitive function after TBI, animal experiments can provide strong evidence. We aimed to conduct a systematic review and meta-analysis involving quantitative analysis of the currently known overall effects of VNS for the treatment of cognitive impairment in TBI based on an unbiased selection of studies; we also present a comprehensive summary and assessment of the mechanisms underlying these effects.

## Methods

This review was conducted in accordance with the Preferred Reporting Items for Systematic Reviews and Meta-Analyses (PRISMA) (Supplementary material 1). The protocol was registered in the International Prospective Register of Systematic Reviews database (CRD42021290797).

### Searching strategy and selection criteria

Two authors independently identified cross-sectional studies describing the effects of VNS, published in peer-reviewed journals before December 2021. Articles were retrieved from six databases (PubMed, PsycINFO, Web of Science, Embase, Scopus, and Cochrane Library) using the medical subject headings(MeSH) and free words union as following: (“vagal nerve stimulation”[Mesh] OR vagus nerve stimulation [Title/Abstract] OR VNS [Title/Abstract] OR iVNS [Title/Abstract]) AND (“cognition” [Mesh] OR cognitive function [Title/Abstract] OR cognitive [Title/Abstract] OR neuropsychological [Title/Abstract] OR neuropsychology [Title/Abstract] OR attention [Title/Abstract] OR orientation [Title/Abstract] OR learn^*^ [Title/Abstract] OR memory [Title/Abstract] OR concentration [Title/Abstract] OR mental-process^*^ [Title/Abstract] OR executive function^*^ [Title/Abstract] OR visuospatial [Title/Abstract] OR language [Title/Abstract] OR intelligence [Title/Abstract] OR intellectual function^*^ [Title/Abstract] OR motor function [Title/Abstract] OR cogniti^*^ [Title/Abstract] OR visual-spatial [Title/Abstract] OR visuo-spatial [Title/Abstract] OR recall [Title/Abstract] OR recognition [Title/Abstract] OR problem solving [Title/Abstract] OR reaction time [Title/Abstract] OR vigilance [Title/Abstract] OR reason^*^ [Title/Abstract] OR psychomotor [Title/Abstract] OR motor [Title/Abstract] OR processing [Title/Abstract] OR planning [Title/Abstract] OR verbal fluency [Title/Abstract] OR inhibit^*^[Title/Abstract]) AND (animal [Title/Abstract] OR mice [Title/Abstract] OR rat [Title/Abstract] OR vivo [Title/Abstract] OR animal model [Title/Abstract]) AND (“Brain Injuries, Traumatic” [Mesh] AND traumatic brain injur^*^ [Title/Abstract] OR TBI [Title/Abstract] OR head injur^*^ [Title/Abstract] OR brain injur^*^ [Title/Abstract] OR brain trauma [Title/Abstract] OR concussion [Title/Abstract] OR concussive [Title/Abstract]). Additionally, the authors searched for relevant studies cited in potentially eligible articles to prevent missing any relevant study.

### Eligibility criteria

The inclusion and exclusion criteria used for screening all the included studies are summarized in Table 1.

**Table 1:** Eligibility criteria.

**Inclusion criteria**	**Exclusion criteria**
Studies in animal models	*Ex vivo* studies, Human studies
Treatment with VNS	Treatment without VNS
Outcomes include behavioral test or pathological changes or other related mechanisms changes	No relevant outcomes reported
Comparison between control group and intervention group	Case studies, cross-over studies
controlled studies with a separate control group	No control group
Independent original data	Not original article, Duplicate data or publications
Articles in English	Articles in other language, No full-text available

### Data extraction and quality assessment

Two reviewers independently performed the initial screening based on the title and abstract and full-text screening of eligible articles for final inclusion. Articles with abstracts that did not provide sufficient information were selected for full-text analysis. Discrepancies were resolved by discussion or consultation with a third investigator.

Two reviewers independently extracted data from the text and Supplementary materials or used the Engauge Digitizer to extract data from figures and tables. We attempted to contact the authors of the included studies through email if the data were not reported or unclear. When an outcome was measured at multiple time points, the data for the time point with the highest efficacy were extracted. Information on the following variables were extracted from each study: (1) publication information (author's name and year of publication); (2) animal information, including animal species, sex, weight, age, TBI model type, or modeling method; (3) intervention information, including experimental group, control group, number of animals per group, VNS type (stimulus type, stimulus location, current density, intervention time, intervention timing, and anesthesia used during the intervention); and (4) outcome assessment, including behavioral approach, area of brain damage, and pathological mechanisms. Discrepancies were addressed by discussion or consultation with a third investigator.

Two investigators independently reviewed the included studies and assessed the risk of bias. The risk of bias was evaluated using the SYstematic Review Centre (SYRCLE) checklist based on the Cochrane Collaboration's RoB tool (29). It consists of 10 items in six main domains: selection bias, performance bias, detection bias, attrition bias, reporting bias, and other sources of bias. Responses to the bias judgments were “yes” for low risk of bias, “no” for high risk of bias, and “UN” for an uncertain level of bias owing to insufficient information. For each item, a “yes” answer was scored as 1 point. We addressed these discrepancies through discussion or consultation with third-party investigators.

### Data synthesis

A meta-analysis was performed using RevMan 5.4. For behavioral testing, brain edema, lesion size, and other variables were expressed as standardized mean differences and 95% confidence intervals (CIs), and a random-effects model was used to account for potential heterogeneity. To avoid recalculating the number of control animals, the sample size of the control group was split into studies using multiple experimental groups and one control group. A Q-statistical test was used to assess heterogeneity, with *P* < 0.05 representing heterogeneity between studies. Values of *I*^2^ were used at 25, 50, and 75% to represent low, moderate, and considerable heterogeneity, respectively.

## Results

### Study inclusion

**Figure 1:**
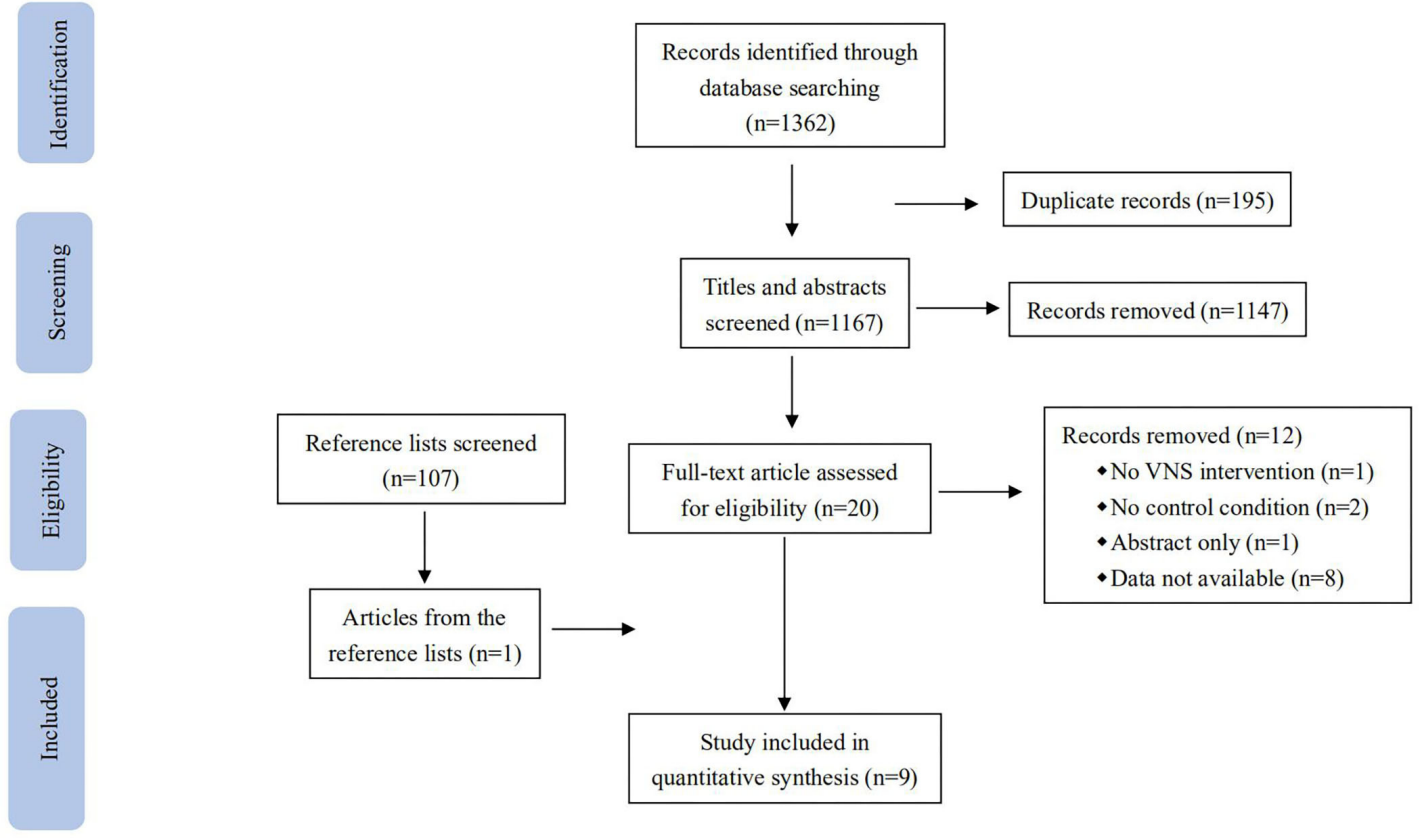
Flow chart for search and study selection.

A total of 1,362 records were identified in the database search (Figure 1): 108 from PubMed, 1,015 from Embase, 22 from Web of Science, 35 from Scopus, 180 from PsycINFO, and 2 from Cochrane. When all searches were combined and duplicate records were removed, 1,167 records remained. After screening the titles and abstracts, 1,147 records that were not eligible for review were excluded. After a full-text review of the remaining 21 records and one record identified from the references, nine studies were identified as being eligible for the predefined inclusion criteria.

### Study characteristics

The included studies were published between 2005 and 2021. The sample sizes of the included studies ranged from 12 to 120. For the species used in each study, seven studies employed rats, one study enrolled mice, and one study involved rabbits (Table 2). Among the studies, males were the most common animals, one study involved an equal number of males and females, and only one study employed females. A weight-drop model of TBI was induced in five studies (30–34), a model of fluid percussion (FPI) TBI was induced in three studies (35–37), and a model of controlled cortical impact (CCI) TBI was induced in one study (38).

**Table 2:** Characteristics of the included studies.

**Included studies**	**Animals**			**Timing of intervention**	**Protocol of VNS**		**Parameters of VNS (Frequency and Current and Pulse width)**	**Comparator (*n =* numbers in each group)**	**Outcomes**		
	**TBI model**	**Sex**	**Weigh**		**Side of VNS (Cervical VN)**	**Duration**			**Behavioral test**	**Pathology**	**Mechanism**
Tang et al. (33)	Weight-drop TBI model, mouse	M	250–300 g	30 min after TBI	Left	One session, 15 min	30 Hz, 1.0 mA, 0.5 ms	TBI+VNS (*n =* 30); TBI+ sham VNS (*n =* 30); sham TBI (*n =* 30)	mNSS↓;	Brain water content↓, necrosis in lesioned brain tissues↓	MDA↓; GSH↑; SOD↑; CAT↑; NLRP3↓, ASC↓, caspase-1↓; Bax↓; Bcl-2↑; IL-1β↓, IL-18↓, IL-6↓,TNF-α ↓
Dong and Feng (31)	Weight-drop TBI model, rat	M, F	250–300 g	1 h after TBI	Left	One session, 15 min	30 Hz, 1.0 mA, 0.5 ms	Sham TBI (*n =* 30); TBI (*n =* 30); TBI+VNS (*n =* 30)	The level of consciousness↑	None	orexin-A↑, OX1R↑
Pruitt et al. (38)	CCI-TBI model, Rat	F	250–300 g	3–4 weeks post-injury	Left	5 weeks; two sessions/day, 30 min/session	30 Hz, 0.8 mA, 0.1 ms	TBI+VNS (*n =* 14); TBI (*n =* 14)	Maximal pull force↑	Lesion size →	None
Zhou et al. (34)	Weight-drop TBI model, rabbit	M	2.0–2.5 kg	1 h after injury	Right	One session, 20 min	5 Hz, 0.8 mA, 5 ms	Blank control (*n =* 4); sham TBI (*n =* 6); TBI (*n =* 9); TBI+VNS (*n =* 8)	None	Brain water content↓	TNF-α↓, IL-1β↓; IL-10↑
Lopez et al. (32)	Weight-drop TBI model, mice	M	20–24 g	None	Right	10 min	-, 2 mA, -	TBI+VNS(N = 4); TBI(*n =* 4); sham TBI(*n =* 4)	None	Neuronal degeneration and vacuolization in the neuropil in neocortex and hippocampal region CA1↓	AQP-4↓
Bansal et al. (30)	Weight-drop TBI model, mice	M	20–24 g	None	Right	10 min	-, 2 mA, -	TBI+VNS(N = 8); TBI(*n =* 8); sham TBI(*n =* 8);	None	None	TNF-α↓, serum levels of Ghrelin↑, Tissue levels of Ghrelin↑
Neese et al. (37)	FPI-TBI model, rat	M	425–475 g	Initiated 24-h post-injury	Left	30minfor the 14-day	20 Hz, 0.5 mA, 0.5 ms	TBI-VN(*n =* 8); TBI (*n =* 8); sham-TBI (*n =* 8)	None	None	GAD_65/67_-like cells in the rostral cerebral cortex ↑, GAD_65/67_-like cells in the hippocampal hilus →
Clough et al. (35)	FPI-TBI model, rat	M	428 g	2 h following FPI surgery	Left	Every 30 min for 48-h duration	20 Hz, 0.5 mA, 0.5 ms	TBI-VNS (*n =* 8); TBI (*n =* 6); sham TBI (*n =* 5)	BWT↑, LPT↑	Brain water content ↓	None
Smith et al. (36)	FPI-TBI, rat	M	425–475 g	2 h following FPI surgery	Left	Every 30 min 30 s stimulation for 14 days	20 Hz, 0.5 mA, 0.5 ms	TBI-VNS (*n =* 15); TBI (*n =* 15); sham TBI (*n =* 15)	SFRT↑, BWT↑, FFT↑, IP↑, LPT↑, MWM↑	Cortical tissue loss →, degenerating neurons in the cerebral cortices, thalamus and basal ganglia →, Hippocampal pyramidal neuron death in the CA-3 of the dorsal hippocampus →, GFAP-stained cells →	None

CCI, controlled cortical impact; FPI, fluid percussion; M, Male; F, Female; TBI, traumatic brain injury; VNS, Vagus nerve stimulation; mNSS, modified neurological severity score; SFRT, skilled forelimb reaching test; BWT, beam walk test; FFT, forelimb flexion test; IP, inclined plane; LPT, locomotor placing test; MWM, morris water maze test; GFAP, glial fibrillary acidic protein; MDA, malondialdehyde; GSH: glutathione; SOD, superoxide dismutase; CAT, catalase; NLRP3, nucleotide-binding domain (NOD)-like receptor protein 3; ASC, apoptosis-associated speck-like protein; Bax, Bcl-2-associated X; Bcl-2: B-cell lymphoma 2; IL-1β, interleukin−1β; IL-18, interleukin−18; IL-6, interleukin−6; TNF-α, tumor necrosis factor-α; OX1R, orexin receptor type 1; IL-10, BBB, blood-brain barrier; AQP-4, Aquaporin 4; GAD, glutamic acid decarboxylase; ↑, significantly increased in animals receiving VNS; ↓, significant decreased in animal receiving VNS; →, no statistically significant difference between groups.

### Intervention characteristics

We observed the current, frequency, intensity, and pulse width of the VNS in all trials and the location of implementation, and the timing and duration of the intervention. Among the included studies, six studies (31, 33, 35–38) presented stimulus locations in the left vagus nerve at the cervical level and three studies (30, 32, 34) were located in the right vagus nerve at the cervical level. The timing of the intervention in most studies (31, 33–37) ranged from 30 min to 24 h at the time of establishing the TBI model. One study (38) intervened 3–4 weeks after establishing the TBI model, and two studies (30, 32) did not mention the timing of the intervention. Five studies (30–34) delivered VNS in only one session, with a range of 10–20 min, and four studies (35–37) ranged from 48 h to 5 weeks of intervention duration. The frequency of VNS in three studies (35–37) was 20 Hz, three studies (31, 33, 38) at 30 Hz, one study (34) at 5 Hz, and two studies (30, 32) did not mention the frequency. VNS currents reported were 2 mA in two studies (30, 32), 1 mA in two studies (31, 33), 0.8 mA in two studies (34, 38), and 0.5 mA in three studies (35–37). One study (34) used a pulse duration of 5 ms, two studies (30, 32) did not report the pulse duration, and the other studies (31, 33, 35–37) used a pulse duration of 0.5 ms. Comparison conditions included sham operation (*k* = 8), TBI with sham VNS (*k* = 1), and TBI with no VNS (*k* = 8).

### Quality assessment

We used the SYRCLE risk of bias assessment tool for animal experiments to assess the risk of bias in the nine included studies (Supplementary material 2). We found that the overall quality of these studies was low, with quality scores ranging from 30 to 60%. None of the studies adequately generated the allocation sequence or described the methods used to conceal the allocation sequence. All included studies had a high risk of performance bias and none of the studies described animals that received the intervention in a blinded manner. Similarly, none of the studies reported that animals were randomly selected for outcome measures, and five studies (31–33, 35, 36) were blinded to the outcome assessor. Seven studies (30, 32, 33, 35–38) reported identical husbandry conditions, whereas two other studies (31, 34) did not describe whether the animals were housed identically during the experiment. Five studies (33, 35–38) reported that the baseline characteristics of each group were similar before VNS. The risks of attrition bias, reporting bias, and other sources of bias were low in all the included studies.

### Neuroprotective effects of VNS in TBI models

#### Behavioral experiments

Behavioral tests were implemented in five of the following nine studies (31, 33, 35, 36, 38) to assess animal motor and cognitive function: Modified Neurological Severity Score (mNSS) (one of nine) (33), Assessment of the consciousness level (one of nine) (31), pull force measurements (one of nine) (38), Beam walk test (two of nine) (35, 36), locomotor placing test (two of nine) (35, 36), skilled forelimb reaching test (one of nine) (36), forelimb flexion test (one of nine) (36), inclined plane (one of nine) (36), and Morris water maze tests (MWM) (one of nine) (36). Vestibular motor function and motor coordination were assessed using the beam walk test (36), a test in which animals escape bright light and enter a dark box by traversing a 120-cm-long elevated beam (2.5-cm wide). Coordination of limb placement during movement was assessed using the locomotor pacing test (35). Animals were allowed to freely traverse the grid for 3 min on an 86 × 55 cm grid surface with 3 × 3 cm^2^ openings. The total number of foot faults and the total number of areas entered were recorded. Two studies have shown improved performance on the beam walk test and locomotor placement test using a 20 Hz, 0.5 mA VNS intervention for 48 hours or 14 days compared to no-VNS (35, 36).

mNSS was used to assess neurological function. It consists of motor (muscle status and abnormal movement), sensory (visual, tactile, and proprioceptive), and balance components. Tang et al. (33) found that the TBI group had higher scores than the sham TBI group. However, VNS treatment significantly reduced mNSS scores (*p* < 0.05), suggesting a protective effect of VNS against neurological injury (33). The level of consciousness was assessed on a scale of I–IV (I–VI levels of consciousness) based on sensory and motor functions. The coma state involves a lack of upright reflex or no response to pain. Dong et al. found that VNS at 30 Hz, 1 mA was applied to the left cervical vagus nerve for 15 min, and 66.7% of rats regained consciousness from a coma. Namely, four rats had reduced activity; six rats had reduced activity with motor incoordination; 10 rats could induce upright reflex and the animals could stand up, compared with only 26.7% (only four rats could elicit righting reflex) in the no-VNS group, indicating that VNS can improve the consciousness of animals (31). Pull force measurements were used to measure the limb force. Pruitt's study showed that VNS enhanced forelimb function and recovery of voluntary pulling strength (38). The skilled forelimb reaching test was performed to assess the animal's forearm locomotion, in which the animal picked up 20 sucrose pellets in succession using only the right forelimb and was scored according to its performance during the pellet retrieval process. The forelimb flexion test was performed to assess the degree of flexion and pronation of the forelimb after injury (36). The rats were placed on a horizontal surface and lifted by holding their tail to observe the degree of flexion of each forelimb. The task of the inclined plane assesses the ability of the animal to maintain its position on an inclined plane. MWM has been used to assess spatial learning and long-term spatial memory in animals (39). It consists of two components: a learning task for spatial acquisition, and a detection task for memory retention. Smith showed that the performance of the animals in the skilled forelimb reaching test (*P* < 0.02), forelimb flexion test (*P* < 0.0001), inclined plane test (*P* < 0.001), and MWM (*P* < 0.0001) that received VNS was significantly better than that of the animals that did not receive VNS (36).

#### Pathological determination

The included studies examined brain tissue edema, lesion size, and neural necrosis in the lesioned tissue; neuronal darkening and degeneration; neuronal vacuolization in the cortical and hippocampal CA1 areas; neuronal degeneration in the cortical, thalamic, and basal ganglia areas; pyramidal necrosis in CA3 in the dorsal hippocampus; and glial fibrillary acidic protein (GFAP) reactive astrocytes cells to observe the pathological features after TBI.

**Figure 2:**
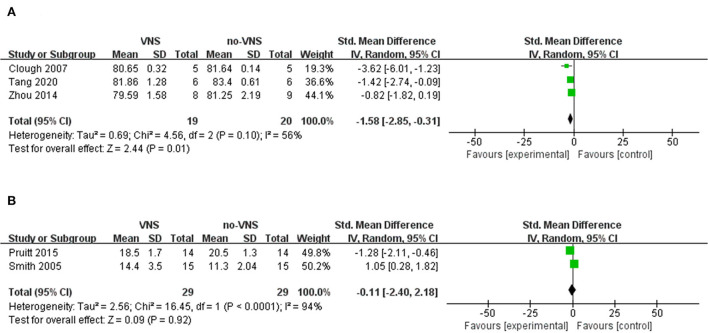
Forest plot for comparison: VNS vs. no-VNS. Brain water content **(A)**, lesion size **(B)**.

Three studies that measured the VNS on brain water content were included in our meta-analysis (33–35). The meta-analysis showed that VNS significantly ameliorated brain edema compared to no-VNS (SMD: −1.58; 95% CI: −2.85, −0.31; *p* = 0.01; *I*^2^ = 56%; Figure 2A). Two studies were included in the meta-analysis to measure VNS based on lesion size (36, 38). The findings showed that VNS did not reduce lesion size compared with no-VNS (SMD: 1.05; 95% CI: 0.28, 1.82; *p* = 0.92; *I*^2^ = 94%; Figure 2B). In two of the included studies, VNS treatment significantly alleviated necrosis of diseased brain tissue and also attenuated TBI-induced neuronal darkening, degeneration, and vacuolation of the neuropil in the cortex and hippocampal CA1 regions (32, 33). In another study, the investigators found no significant differences in cortical lesion size, degenerating neurons, hippocampal CA-3 cell number, and GFAP-stained cells (36).

### Neuroprotective mechanisms of VNS in TBI models

#### Oxidative stress

After TBI, there is an increase in oxidative stress markers such as malondialdehyde (MDA) and a decrease in antioxidant defense enzymes including glutathione (GSH), superoxide dismutase (SOD), and catalase (CAT) in the brain, and this imbalance between oxidants and antioxidants can lead to neurological dysfunction and death. The study showed that VNS delivered to the left vagus nerve at the cervical level at 30 Hz, 1 mA for 15 min significantly reduced the level of MDA and increased the levels of GSH, SOD, and CAT compared with those in the no-VNS group (33).

#### Neuronal apoptosis

In addition, the Bcl-2-associated X (Bax) protein promotes apoptosis, whereas the B-cell lymphoma 2 (Bcl-2) protein inhibits apoptosis induced by various injuries. The study reported that the expression of Bax was significantly increased and that of Bcl-2 was decreased in the TBI model. However, Tang et al. found that after 15 min of VNS intervention, the expression level of Bax decreased and that of Bcl-2 increased (33). VNS may contribute to anti-oxidative stress by controlling the oxidant-antioxidant balance to inhibit protein oxidation and DNA cleavage (19). However, whether it may regulate the production or degradation of reactive oxygen species (ROS) still needs further exploration.

#### Neuroinflammation

**Figure 3:**
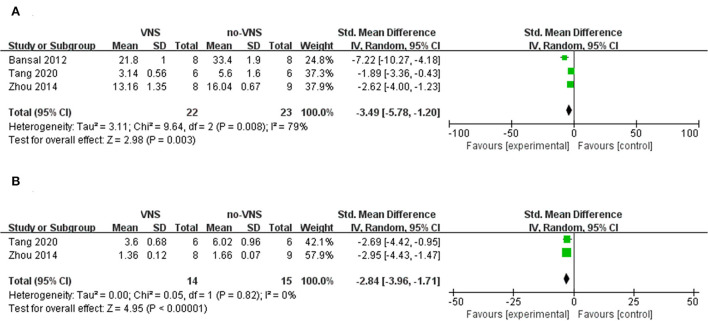
Forest plot for comparison: VNS vs. no-VNS. TNF-a **(A)**, IL-1ß **(B)**.

After trauma, oxidative stress induces the release of pro-inflammatory factors and exacerbates inflammation through the activation of nuclear transcription factor-κB (NF-κB). Nucleotide-binding domain-like receptor protein 3 (NLRP3), a downstream mediator of NF-κB, is composed of NLRP3, apoptosis-associated speck-like protein (ASC), and caspase-1 molecules, which are members of the family of recognition receptors involved in the innate immune response and play a role in TBI. Upon its activation, procaspase-1 is cleaved to caspase-1, triggering the release of interleukin (IL)-1β and IL-18. Tumor necrosis factor (TNF-α), IL-1β, IL-18, and IL-6 are pro-inflammatory mediators that induce inflammation, modulate immunity, and promote inflammatory cascades, leading to secondary brain tissue lesions, secondary edema, and brain cell necrosis (40, 41). IL-10 is an anti-inflammatory factor that exerts an inhibitory effect on immune inflammatory responses and tissue damage remodeling and repair (42). Three studies showed that compared with no-VNS, VNS treatment reduced the expression level of TNF-α (SMD: −3.49; 95% CI: −5.78, −1.2; *p*=0.003; *I*^2^ = 79%; Figure 3A), but did not reduce the expression level of IL-1β (SMD: −2.84; 95% CI: −3.96, −1.71; *p* < 0.00001; *I*^2^ = 0%; Figure 3B).

The weight-drop brain injury model was used in all three studies, and the timepoint of the VNS intervention was between 30 min and 1 h after brain injury. One study (33) performed a 15-min VNS at 30 Hz, 1 mA delivered to the left cervical vagus nerve of the animal. The other two studies (30, 34) intervened in the right cervical vagus nerve of the animals at 5 Hz, 0.8 mA for 20 min, and at 2 mA for 10 min, respectively. As shown above, both VNS can reduce brain tissue or serum TNF-α levels by stimulating the left or right vagus nerve for 10–20 min in animals with weight-drop brain injury. It is not completely clear how VNS inhibits neuroinflammation and reduces inflammatory factors, such as TNF-α, but studies have found that VNS can activate alpha7 nicotinic acetylcholine receptor (α7nAChR) on microglia to regulate NF-κB, JAK2/STAT3, PI3K/AKT signaling pathways to inhibit the expression of TNF-α, IL-1, IL-6, and other inflammatory factors (33, 43, 44). In addition, Vishal Bansal et al. found that VNS can upregulate the expression of brain gut peptides, which can reduce the release of TNF (30). Li et al. also found that VNS can play an anti-inflammatory role and reduce TNF in brain injury by upregulating the expression of peroxisome proliferator-activated receptor γ (PPARγ) (45).

#### Blood-brain barrier

Post-traumatic brain edema is a complex physiological process involving the disruption of the blood-brain barrier (BBB). Aquaporin 4 (AQP-4) is a unique bidirectional water channel protein found in astrocytes, which are endothelial cells that reinforce the BBB. In TBI, perivascular AQP-4 is increased, and this increase is thought to play a role in brain edema. The study found that VNS stimulated the right cervical vagus nerve of mice at 2 mA for 10 min, which significantly reduced vascular permeability (VP) (*p* < 0.05) and decreased the upregulation of AQP-4 (*p* < 0.001) compared with TBI alone (32). The inflammatory cytokines are thought to be mediators of BBB permeability, which can disrupt the BBB. VNS attenuates BBB disruption after TBI, which may be associated with the downregulation of AQP-4 and inflammatory factors. It suggests that the regulation of AQP-4 may be another neuroprotective effect of VNS.

#### Gamma-aminobutyric acid

Gamma-aminobutyric acid (GABA) is produced in neurons that regulate cortical and thalamocortical circuits; transmit sensory information; and play a role in coordinating motor function, attention, and other aspects. Regulation of GABAergic signaling involves the synthesis of GABA from glutamate by glutamic acid decarboxylase (GAD). After the injury, the loss of GABA-producing cells disrupts the balance of excitation and inhibition, leading to further cellular injury. Neese et al. found that VNS in 20 Hz, 0.5 mA for 14 days significantly increased the expression of GAD65/67-like cells as two isoforms of GABAergic cells in the rostral cerebral cortex (*p* < 0.05) compared with no-VNS. However, no significant differences were observed in the hippocampal hilus (37). This is consistent with the results of other studies (46, 47). Recent fMRI studies have confirmed that tVNS activates the NTS by stimulating afferent fibers, resulting in enhanced GABA release (48–50).

Dong et al. found that VNS upregulated orexin-A and orexin receptor type 1 (OX1R) expression in the prefrontal cortex to recover consciousness from TBI-induced coma. Orexin-A is one of the most important neurotransmitters in the ascending reticular activating system and is involved in the consciousness and sleep-wake cycles. OX1R regulates feeding behavior, energy balance, neuroendocrine activity, and the sleep-wake cycle (31). This may be associated with VNS activating vagal afferent fibers that project *via* the nucleus accumbens to many brainstem regions, including the ventricles, parabrachial nuclei, thalamus, basal forebrain, hypothalamus, and cerebral cortex, resulting in activation of the ascending reticular activating system, which plays a key role in promoting wakefulness. At the same time, VNS significantly increases norepinephrine, 5-HT, and dopamine, all neurotransmitters in the central nervous system that may have a pro-arousal effect (51, 52).

## Discussion

The systematic review showed that VNS improved performance in behavioral tests and locomotor placing tests, whereas it reduced brain edema and the expression level of TNF-α in the brain tissues.

Traumatic brain injury refers to direct or indirect brain injury caused by external violence, usually resulting in a series of functional and other pathological changes in the brain. Despite the fact that treatment in the acute phase of TBI has greatly improved the survival rate of patients, there are still associated impairments, in which cognitive impairment is the main clinical feature. Cognitive impairment is often characterized by deficits in attention, memory loss, and reduced executive ability. In more than 50% of patients with TBI, there is persistent impairment of residual cognitive function and a link between this and neurodegenerative disease, even to the point of developing dementia (53–55). However, the mechanisms of cognitive impairment after TBI are not yet fully understood and there are no particularly effective therapeutic approaches. VNS has been approved by the U.S. Food and Drug Administration as an alternative treatment for refractory epilepsy, refractory depression, cluster headache, and migraine (11, 56, 57). The use of VNS has been extended to treat a broader range of brain disorders, such as Parkinson's disease, Alzheimer's disease, stroke, and TBI (25, 58). To better understand the effects of VNS on behavioral and pathological characteristic changes and therapeutic mechanisms after TBI, investigators have conducted preclinical studies using animal models that mimic the neuropathological changes and cognitive deficits associated with TBI. There is some evidence that VNS can protect neurological function and cognitive function after TBI (25, 58). A meta-analysis based on a systematic review is a powerful tool to evaluate the results of these trials as a way to validate the potential of VNS as a treatment for cognitive impairment after TBI and to provide evidence for its use in the clinical setting. All included studies investigated the therapeutic effects of VNS on TBI. They treated three animal models of TBI, namely, the weight-drop model, FPI, and CCI, with or without VNS, and observed or examined changes in the behavioral performance and pathological characteristics of the animals. In most of these nine studies, comparisons of outcomes showed significant effects, but statistically insignificant effects were also observed.

Various methods were used to obtain the TBI model in the enrolled studies. These include the weight-drop TBI model, which uses the gravitational force of free fall to produce focal or diffuse brain injury; the CCI-TBI model, which produces focal brain injury by a computer-assisted controlled acceleration of a rod against a surgically exposed dura; and the FPI-TBI model, which consists of a pendulum striking a piston at the end of a fluid-filled tube to produce focal or diffuse brain injury (59). VNS with a frequency of 30 Hz and a current of 1 mA was used to target the left cervical vagus nerve in weight-drop TBI animals by Tang et al. and Dong et al. (31, 33). Both found that VNS improved neurological function and level of consciousness in the animals. In other weight-drop TBI model studies (30, 32, 34), VNS at 0.8 mA and 2 mA were used to intervene in the right cervical vagus nerve of animals, and although none of them reported results related to cognitive function, they found that VNS decreased brain tissue edema, reduced hippocampal neuronal apoptosis, and inhibited neuroinflammation after TBI. VNS with 30 Hz, 0.8 mA VNS was administered to rats with CCI-TBI for 30 min each time, twice daily, for 5 weeks VNS, and the animals showed a significant increase in paw pull, but no cognitive function was reported (38). Alternatively, the investigators administered VNS at 20 Hz, 0.5 mA to FPI-TBI rats for several consecutive repetitions and showed that VNS improved not only the neuromotor function of the animals but also the cognitive function (35–37). Overall, although the included studies were conducted using different TBI models and different parameters and duration of VNS, VNS improved motor function in TBI, but there is less evidence for the effect on cognitive function, and more studies are still needed to confirm this.

Vagus nerve stimulation includes invasive VNS (iVNS) and non-invasive VNS (nVNS) (11). iVNS, which involves implanting electrodes at the left cervical vagus nerve and thus stimulating the vagus nerve, is somewhat limited in clinical practice due to its invasive. nVNS is a new non-invasive method of stimulation targeting the superficial auricular and cervical branches of the vagus nerve. nVNS does not involve the risks associated with surgical electrode implantation and has the advantage that treatment can be easily stopped or removed if the patient does not respond intentionally to VNS (19). Therefore, there will be potentially unsatisfactory effects in patients receiving nVNS due to lower adherence. Our review of the literature revealed that nVNS has been widely used in clinical studies to improve cognitive function and less frequently in animal models. A few studies found that nVNS improved the Coma Recovery Scale—Revised (CRS-R) especially in motor function in disorders of consciousness (DOC) patients after TBI (58). However, iVNS was almost always used in studies of animal models of TBI.

Cognitive impairment in TBI results from both primary and secondary injuries (60). In primary brain injury, patients present with different forms of laceration, contusion, epidural hematoma, subarachnoid hemorrhage, subdural hematoma, or diffuse axonal injury, which can cause destruction of brain structures or deformation of tissues, leading to a series of pathological changes, such as degeneration or death of neurons, cellular excitotoxicity, inflammation, the release of oxygen free radicals, and elevation in the intracranial pressure due to brain edema. Long-term pathological changes caused by primary neuronal injury can lead to secondary brain injury, including neuroinflammatory responses, neurogenic fiber tangles, β-amyloid peptide deposition, apoptotic necrosis, and lipid oxidative stress damage, all of which can lead to varying degrees of cognitive impairment. Therefore, interventions for cognitive impairment in TBI may need to address these pathological features (61).

This review showed that VNS promoted recovery from TBI, and its effect was attributed to the reduction of brain edema, but it did not change the size of the pathological structures. In addition, VNS attenuated oxidative stress and apoptosis in the cortex after TBI, reduced the level of inflammatory factors in the brain tissue, and attenuated cerebral VP, while VNS had an overall protective effect on GABAergic neurons. This finding is consistent with those of previous reports that the mechanism of VNS in brain disease involves both anti-inflammatory and central nervous system mechanisms. After TBI, the interaction among inflammation, oxidative stress, BBB, and brain edema causes secondary damage to the brain (32, 62). In TBI, inflammation is a protective response to external stimuli in the acute stage. However, excessive inflammation can lead to, or accelerate, the development of various brain diseases. ROS released by neuroinflammation in TBI generates oxidative stress, which can induce peroxidation of membrane lipids and damage the BBB (63). Increased permeability of the BBB is usually involved in inflammation-associated neurodegeneration (64, 65). In addition, disruption of BBB integrity and inflammatory responses contributes to brain edema after TBI (66). Studies have shown that the vagus nerve is involved in inflammatory injuries. Additionally, VNS may alleviate clinical symptoms by suppressing systemic or local inflammation. Although the precise anti-inflammatory mechanism of VNS is not fully understood, it has been shown to be related to the regulation of cytokines released peripherally from immune cells, microglial status, and alterations in the permeability of the BBB. In addition, VNS promotes the release of neurotransmitters, such as acetylcholine (Ach), NE, GABA, and γ-aminobutyric acid from the locus coeruleus (LC), accelerating the neurological remodeling of the central nervous system (19, 32, 67). It is worth being concerned about the various posttraumatic symptoms that can occur after TBI (4). In a review on epilepsy after TBI, it was mentioned that the altered GABA levels in the CNS after TBI and neuroinflammation may be underlying mechanisms leading to seizures. Regulating neurotransmitters, directly and indirectly, may be a novel target for post-traumatic epilepsy (PTE) (68, 69). Thus, VNS may modulate neurotransmitters, such as GABA, reduce glutamate-induced toxic effects, inhibit JNK signaling, and treat PTE.

To the best of our knowledge, this is the first systematic review of RCTs investigating VNS that was applied to TBI animals, thus providing a comprehensive synthesis of the evidence. Nevertheless, some limitations should be addressed. Different outcomes were used in the included studies, resulting in an analysis that was not suitable for publication bias, so the present results may have been influenced by the small sample of studies. Meanwhile, certain challenges remain in this area of research. First, existing studies have fewer behavioral assessments of cognitive function, and there is insufficient evidence that VNS improves cognitive function after TBI. Second, studies have used more scattered assessment methods and have not conducted subgroup analyses of VNS parameters and intervention durations to determine the optimal stimulus for VNS. Some studies have suggested that the effect of VNS stimulation is influenced by stimulation frequency, intensity, and duration (19). Third, most of the studies applied VNS within 24 h after TBI, and fewer studies observed the effect of VNS intervention in the chronic phase of TBI. Cognitive function in TBI has different mechanisms at different stages, and future studies are needed to test the cerebral protective effect of VNS in the acute and chronic phases of TBI. Finally, despite the various changes observed after VNS, it is difficult to distinguish them from the original effects of VNS owing to the multiple mechanisms involved in cognitive impairment after TBI and the complex innervation of the VN. The exact mechanisms underlying the therapeutic effects of VNS are still not fully understood. Therefore, we should pay more attention to these issues in future studies on VNS.

In conclusion, the present study suggests that VNS may reduce brain injury after TBI by inhibiting oxidative stress and inflammation, reducing BBB permeability, and modulating neurotransmitters. VNS for the treatment of cognitive dysfunction after brain injury has achieved initial results in animal experiments and clinical applications. The promising results of VNS as an autonomic nerve stimulation technique applied to brain injury not only show the application prospects of new technology, but more importantly, provide new thinking that the vagus nerve plays an important role in cognitive function. A full understanding of the interaction between the vagus nerve and brain cognition will require a further investigation of the mechanism of VNS, biological markers, and optimization of stimulation parameters to maximize the efficacy of VNS in brain-injured patients.

## Data availability statement

The original contributions presented in the study are included in the article/Supplementary material, further inquiries can be directed to the corresponding author.

## Author contributions

HZ and YQ conceptualized and wrote the study. C-lL, Y-xY, and JD performed the study selection. HZ and YZ prepared the manuscript. All authors contributed to the writing of the article and approved the submitted version.

## Funding

This study was supported by the National Key R&D Plan (2017YFC1308504) and the Youth Innovation Research Project of Sichuan Provincial Medical (Q20036).

## Conflict of interest

The authors declare that the research was conducted in the absence of any commercial or financial relationships that could be construed as a potential conflict of interest.

## Publisher's note

All claims expressed in this article are solely those of the authors and do not necessarily represent those of their affiliated organizations, or those of the publisher, the editors and the reviewers. Any product that may be evaluated in this article, or claim that may be made by its manufacturer, is not guaranteed or endorsed by the publisher.
