# Supplementary material for: Effects and neuroprotective mechanisms of vagus nerve stimulation on cognitive impairment with traumatic brain injury in animal studies: A systematic review and meta-analysis
Source: Front Neurol. 2022 Sep 27;13:963334. doi: 10.3389/fneur.2022.963334 (PMC9551312; doi:10.3389/fneur.2022.963334)
Supplement: Supplementary file 2 [file Table_2.docx]

| Table S2. Quality assessment of included studies. | | | | | | | | | | | |  |
| --- | --- | --- | --- | --- | --- | --- | --- | --- | --- | --- | --- | --- |
| Included studies | Selection bias | | | Performance bias | | Detection bias | | Attrition bias | Reporting bias | Other sources of bias | Quality score (“yes” items) | Quality score (%) |
|  | Sequence generation | Baseline characteristics | Allocation concealment | Random hosing | Blinding | Random outcome | Blinding | Incomplete outcome data | Selective outcome reporting |  |  |  |
| Tang, 2020 | U | Y | U | Y | N | U | Y | Y | Y | Y | 6 | 60% |
| Dong, 2018 | U | U | U | U | N | U | Y | Y | Y | Y | 4 | 40% |
| Pruitt, 2015 | U | Y | U | U | N | U | U | Y | Y | U | 3 | 30% |
| Zhou, 2014 | U | U | U | Y | N | U | U | Y | Y | Y | 4 | 40% |
| Lopez, 2012 | U | U | U | U | N | U | Y | Y | Y | U | 3 | 30% |
| Bansal, 2012 | U | U | U | U | N | U | U | Y | Y | Y | 3 | 30% |
| Neese, 2007 | U | Y | U | U | N | U | U | Y | Y | U | 3 | 30% |
| Clough, 2007 | U | Y | U | U | N | U | Y | Y | Y | U | 4 | 40% |
| Smith,2005 | U | Y | U | U | N | U | Y | Y | Y | U | 4 | 40% |

Y: low risk of bias; N, high risk of bias; U, unclear risk of bias.
